# The attitudes of homeless women in London towards contraception

**DOI:** 10.1017/S1463423619000665

**Published:** 2019-09-12

**Authors:** Pooja Shah, Tamar Koch, Surinder Singh

**Affiliations:** 1 Department of Primary Care and Population Health, University College London (UCL), London, UK; 2 Clinical Teaching Fellow & GP, Department of Primary Care and Population Health, University College London (UCL), London, UK; 3 Senior Lecturer in General Practice & GP, Department of Primary Care and Population Health, University College London (UCL), London, UK

**Keywords:** contraception, homeless, London, vulnerable, women

## Abstract

**Aim:**

To gain a clearer understanding of the attitudes of homeless women towards contraception in central London.

**Background:**

Homeless women are more vulnerable to sexually transmitted infections and unwanted pregnancies. This makes it imperative to address the health needs of this population. Evidence regarding the obstacles homeless women face when using contraception and accessing sexual/reproductive care is sparse, and almost non-existent in the United Kingdom (UK). American research has identified past experiences of women suffering side effects and their fear of serious health risks as deterrents of sustained contraceptive use among this population.

**Method:**

This study used convenience sampling and semi-structured face-to-face interviews. During the interview, a topic guide was used to ensure data relevant to the study aim were being collected. In total, 14 English-speaking women, previously street homeless and/or living in temporary accommodation from two homeless shelters located in central London, were interviewed.

**Findings:**

In summary, the results suggest this group of study respondents find ongoing access to advice on contraception services difficult largely because of their homelessness. This pre-eminent factor alongside their vulnerability inevitably means that other issues take precedence on a daily basis. Furthermore, issues such as individual choice of contraception and the perceptions of this group of women to health professionals ultimately determine whether women receive the services they need. Bearing in mind the paucity of studies in this area of homelessness, the results point to the need for more research and to the allied question ‘how is it best to provide contraceptive services to those women who find themselves homeless?’

## Introduction

Due to the transient nature of the homeless population, it is very difficult to calculate their number at any one time as the term ‘homeless’ covers a multitude of categories such as those of no fixed address, street homeless, sofa-surfing, temporary homeless and those who cannot afford their current accommodation (Healthy London Partnership, 2017). The number of hidden homeless –people who are homeless and not receiving support and are therefore not known about – is unknown, which makes it difficult to calculate the total population. It is estimated that there are 13 times more hidden homeless than those sleeping rough in London (London Assembly Housing Committee, 2017). Nevertheless, evidence suggests that street homelessness has risen over recent years along with an upward trend in the use of temporary accommodation (Homeless Link, 2014).

In 2010, Homeless Link published data looking at the health of the homeless population (Homeless Link, 2014). Results showed that the homeless experience some of the worst health problems in society partly because they are living on the street or in temporary accommodation. The report also stated that a number of homeless people are not receiving help with their health problems (Thomas, 2011; Homeless Link, 2014), making them more vulnerable to a vast number of illnesses, including poor sexual health, compared to the general population.

It is reported that the homeless are at an increased risk of sexually transmitted infections (STIs) and unwanted pregnancies (DH and Cross Government, 2013). Studies in North America have shown that approximately 73% of pregnancies among homeless women were unintended at the time of conception and STIs among homeless women were as high as 60% (Gelberg *et al.*, 2001). These could potentially be reduced by removing the obstacles homeless women face when attempting to access medical services (Wenzel *et al.*, 2001). An American study reported that accessing women’s health services appeared to be an inconvenience for some homeless women due to long waiting times, cost and transportation issues (Gelberg *et al.*, 2004).

There is some pertinent literature regarding the factors that influence a homeless woman’s use of contraception in the American population. As the United States (US) has a different health care system compared to the United Kingdom (UK), it is unlikely the American studies accurately reflect the barriers homeless women in the UK encounter. The UK has a long tradition of trying to provide primary care to otherwise vulnerable and marginalised groups (Gill and Wright, 2014). Theoretically, anyone can access contraceptive services through outreach programmes and primary care, though not all will provide the service. Nonetheless, there is a lack of research specifically into the urban UK population, which is what this study aims to explore.

## Methods

The study was undertaken using comprehensive, semi-structured interviews. Interviews were carried out at two homeless shelters in different parts of central London. Participants were recruited from each of these homeless shelters. Women between the age range of 18–55 years who had been street homeless in the past and/or living in temporary accommodation and English-speaking were included in the study. Recruitment was undertaken by direct invitation at the first hostel through the hostel manager, and a drop-in session at the second hostel where women were made aware of the study by word of mouth through hostel staff members. Interviews only commenced once verbal and written consent was obtained.

A semi-structured interview was undertaken using a topic guide to ensure data relevant to the study aim were collected (see topic guide). The topic guide included a set of open-ended questions and prompts for potential conversation topics based on existing literature and thoughts from the authors. Interviews were carried out within the shelter in which the participant was resident. At the end of the interview, participants were given a £5 food voucher as a token of thanks. Interviews were conducted between February and March 2016. Interviews were audio recorded and transcribed verbatim. The interviewer made observational and reflective notes immediately after each interview (Spradley and McCurdy, 1980).

A thematic analysis approach was employed (Pope *et al.*, 2000), led by the interviewer soon after the first set of interviews. Initial themes were identified and derived from the data. An iterative approach was employed to enhance the data collection and analysis process. As interviews progressed, there was constant comparison of the data to the initial themes identified and new themes were added where necessary. All transcripts were then reassessed in search of any evidence that may have been missed. To increase the reliability of the findings, two additional readers (T.K. and S.S.) read through a transcript of an interview independently of the principal researcher to identify themes, which was then compared and searched for consistency.

## Findings

There were a total of 14 participants and the median age was 27 years. Data analysis resulted in three core themes: ‘homeless and vulnerable’, ‘individual factors’ and ‘health professionals’.

### Homeless and vulnerable

The majority of the respondents were deemed vulnerable and recognised that their use of contraception was important in preventing a pregnancy during this period in their life:

*“This right moment in time, when your mind frame is about just trying to survive, you don’t want to have a baby at this time”*.

(P10, 30-year-old)

Being homeless, participants faced unique and difficult circumstances, partly explaining their use of contraception:

*“I do know a lot of homeless women turned to street prostitution”*.

(P7, 27-year-old)

*“If you don’t have a place to sleep, any man who have a place to sleep can just call you and say you can move in with me. Say if I don’t have a place to sleep, I have to go. Then the man will take advantage because you don’t have a place”*.

(P13, 38-year-old)

Many participants stated that thinking about contraception would not be at the forefront of their mind due to their lack of stability and transient lifestyle.

*“I think when you are homeless you’re in such a dark place… you don’t do anything”*.

(P1, Unknown age)

*“Once you’re homeless you don’t think to go to a hospital or a GP or you don’t think … Normal daily routine is missing for you. You’re like all over the place really”*.

(P10, 30-year-old)

Most participants stated that accessing contraception was not their upmost priority.

*“…thinking about buying contraception when you got to pay rent, do washing, buy food… I don’t think people would really bother [with contraception]”*.

(P1, Unknown age)

Participants explained that homeless women do have the knowledge of where to access contraceptive services hence potentially reducing their vulnerability (so long as they could get to the clinic).

*“It’s not because people are on the street. They are clever. People are intelligent. And I don’t think it’s about information. It’s about responsibility”*.

(P14, 31-year-old)

*“…people think that because people are homeless they are not educated. Some of them have gone to school, some of them went to uni… I think the homeless is not the homeless of 40 years ago… So, I know where to go”*.

(P14, 31-year-old)

Local primary health care providers did not offer contraceptive services hence women had to travel a great distance away, which had a great impact on participants’ utilisation. Services that were located close by and convenient, for example, in hostels, were preferred by all participants.

*“They’re in Tottenham and that’s a trek, especially if you haven’t got any money… that’s a long way to walk”*.

(P2, Unknown age)

### Individual factors

Participants’ use of contraception depended on a number of personal factors.

Some women’s utilisation of contraception was partly governed by their religious background and culture, of which conflicting views were seen:

*“I had to get on contraception with being Muslim, you don’t want to get pregnant before you’re actually married”*.

(P10, 30-year-old)

*“God almighty made us to have babies…So why are we blocking it? …By right we should not be blocking whatever has been in us to produce”*.

(P13, 38-year-old)

Numerous participants felt uncomfortable when accessing contraception.

*“It’s quite embarrassing when you go in there and you go and ask for it… I’m 27 but it still does get embarrassing”*.

(P7, 27-year-old)

Participants’ choice of contraception varied for a number of different reasons.

A reluctance to try long-acting reversible contraceptives (LARCs) stemmed from the concern about having a foreign object inside their body:

*“I’m also scared of having, knowing that there’s something inserted inside of me 24/7… I find that weird”*.

(P4, 22-year-old)

However, others were in favour of using LARCs:

*“I think it’s a lot easier [using LARCs]… because sometimes you don’t remember…because things are always going on”*.

(P2, Unknown age)

Nonetheless, some participants recognised that accessing LARCs would be more of a hassle compared to accessing condoms:

*“… if I were to weigh out to use a condom or just go through all that long process I’d rather use a condom to prevent me from going to the clinic”*.

(P9, 26-year-old)

A hesitancy to use some methods of contraception arose from having previous bad experiences such as side effects:

*“I got quite ill from being on the pill, I don’t think I would go back to using it”*.

(P7, 27-year-old)

Some participants’ partners were involved in the decision-making process about what contraceptive method to use:

*“At the beginning of my relationship with my boyfriend…he was a bit iffy about using condoms at first”*.

(P7, 27-year-old)

All participants recognised the benefit of having access to free contraceptive services and the availability of highly effective contraceptive methods, which encouraged them to seek out and utilise these services:

*“…knowing you can get the services for free, it’s actually really useful and you feel safe”*.

(P11, 21-year-old)

Mixed views were seen among participants about long waiting times at a particular clinic. Some participants found this a deterrent, while others were not so bothered by it.

*“I mean when you are homeless… you’ve got a lot of time on your hands and if you’ve been waiting in a waiting room, you’re indoors aren’t you?”*.

(P2, Unknown age)

### Health professionals

Mixed views were seen among participants about building a professional relationship with those offering contraceptive services:

*“They kinda got to know me as well… it just feels like you’ve got a trusted relationship with them”*.

(P7, 27-year-old)

Others were more comfortable discussing contraception with someone they were not as familiar with:

*“… you’re slightly embarrassed maybe you want to see someone you’ve never seen before”*.

(P2, Unknown age)

Many women indicated that they were not made aware of what contraceptive methods were available to them by health professionals when they visited clinics, thus having an impact on what contraceptive method they used:

*“In my GP… I just went in and asked her what I wanted and they didn’t even say if I wanted A, B, C or D. I just asked for what I wanted, got the prescription and went”*.

(P7, 27-year-old)

Participants highlighted that an unwelcoming health care setting discouraged them to open up about problems they were facing:

*“I think they should be more welcoming in clinics… its like a system. Get in, get out kind of thing. Clock in, clock out”*.

(P10, 30-year-old)

See Table 1.

**Table 1.** Participant information

Participant number	Age (years)	Previously street homeless
P1	Unknown	No
P2	Unknown	Yes
P3	Unknown	No
P4	22	No
P5	53	Yes
P6	26	Yes
P7	27	Yes
P8	27	No
P9	26	No
P10	30	Yes
P11	21	Yes
P12	25	No
P13	38	Yes
P14	31	Yes

## Discussion

This small study provides an insight into understanding the attitudes of homeless women towards contraception in central London. Themes identified included ‘homeless and vulnerable’, ‘individual factors’ and ‘health professionals’.

The theme ‘homeless and vulnerable’ explored issues around contraception that were previously under-reported in the UK such as being in a vulnerable position while homeless, involvement in prostitution, lack of mental stability and disjointed lifestyle, all of which affected participants access to and use of contraception.

It has been long known that the state of homelessness means an increased risk to health on account of vulnerability and the link with substance abuse and mental health concerns are well documented (Gelberg *et al.*, 2008).

Traditionally, women choose contraception based on several factors and the participants in this study were no different. This study looked at a particularly disadvantaged group of women – previously street homeless and those in shelters – where factors such as convenience and cost are crucial. Some of the findings within this theme ‘homeless and vulnerable’ are consistent with many of the results identified by other studies (Killion, 1998; Gelberg *et al*., 2002; Gelberg *et al.*, 2004; Saver *et al.*, 2012) where factors such as transportation costs, distant location of health clinics and long waiting times were genuine reasons why services were not utilised. Attempts to bring health care professionals to where people are temporarily housed have been tried – both in the UK (GP services for rough-sleepers in Lewisham, 2018) and in Scotland (FRSH, 2018).

However other findings within the themes, knowledge of where to access contraception and cost of contraception appeared to contradict previous findings from the US. Killion’s ethnographic study in the US (1998) reported that lack of knowledge of local services limited homeless women’s use of contraception and placed them in more of a vulnerable position. Gelberg *et al.* (2004) reported, again from the US, that contraception could be accessed so long as women were resourceful, confident and could plan their health needs, though there was a cost implication. In contrast, our study highlighted that participants had sufficient local knowledge of where to access contraceptive services. Additionally, many participants in this study acknowledged that free contraceptive services are a great benefit – clearly evidence shows that services are being accessed readily and perhaps regularly by some women.

The theme ‘Individual factors’ explored participants’ considerations when choosing which contraceptive method to use. Past experiences and perceived intrusiveness of contraception emerged as factors that participants considered when choosing a contraceptive method. Some of these findings coincided with those reported by Kennedy *et al.* (2014).

Stigma and lack of respect shown by health care professionals was often mentioned as a drawback by participants in the study conducted by Gelberg *et al.* (2004). Similar findings are also reported in this study under the theme ‘Health Professionals’ whereby some participants were not made to feel welcome upon using such services. A number of women said they felt embarrassed when attending the clinic and were not made aware of key information during the consultation.

Overall, as illustrated above, this study concurs with findings from previous studies, though it is important to acknowledge that this is an area which is under-researched in the UK.

## Strengths and limitations

This study addresses an issue where evidence is sparse, and almost non-existent in the UK. This is one of the first studies which aimed to explore and examine some of the reasons why contraception is a significant issue in a group of women whose needs are understated, under-recognised and often dismissed in an urban setting, such as central London. The participants were recruited from two homeless shelters in London; therefore, it may be useful to recruit homeless participants from other settings for future studies, for example, the street. However, due to the small scale of this study, generalisations cannot be made about other groups of homeless women or rough sleepers.

## Conclusion

Women find themselves with extra burdens when homeless – for example, the care of themselves and children, and in some instances how to access contraceptive and sexual health services. In this London study, albeit limited in size the women could access contraception but that personal factors and choice ultimately determined the type chosen. One factor, perhaps hitherto unanticipated, was the attitude of professionals in the clinics – some of which were negative and unwelcoming. There is a danger that some of these attitudes could be perceived as further obstacles to care for this group of vulnerable women.

It should be noted that these issues are not the sole domain of sexual health services only. General primary health care services and local charity initiatives working with the homeless and rough-sleepers population – including specific outreach programmes – should all aspire to provide access to services, including contraceptive services, and reduce health inequalities.

This could be achieved by providing user-friendly services within hostels and temporary accommodation where homeless women are resident. The delivery of the services could be improved by providing staff training to make the services more patient friendly to increase the uptake of the services. The findings of this small study prompt further enquiry into an area that is clearly of fundamental importance to homeless women.

## Supplementary material

For supplementary material accompanying this paper visit https://doi.org/10.1017/S1463423619000665. click here to view supplementary material
