# Supplementary material for: The attitudes of homeless women in London towards contraception
Source: Prim Health Care Res Dev. 2019 Sep 12;20:e131. doi: 10.1017/S1463423619000665 (PMC6749551; doi:10.1017/S1463423619000665)
Supplement: Supplementary file 1 [file S1463423619000665sup001.docx]

**Topic Guide:**

Introduction:

- Welcome the participant and interviewer introduces themselves.
- Explain clearly the purpose of the study and format of the interview e.g. length, types of questions that will be asked.
- Ensure the consent form is completed, participant signature is present and form has been received.
- Confirm verbally that the participant is willing to partake in this research study.
- Reassure the participant that anything disclosed in the interview is entirely CONFIDENTIAL, will remain entirely anonymous and will not be judged by the interviewer.
- Ask the participant to be as honest and open about the topic concerned, solely for the purpose of data collection for the study.
- Tell the participant that they are not obliged to answer every question and they are free to withdraw from the study at any time they wish.
- Ask the participant if it is okay to commence the interview.

TAPE ON

Say INTERVIEWEE NUMBER

The number of unwanted pregnancies in homeless women appears to be high. Why do you think that is?

Prompts:

Do you face any problems when you try to access contraceptive services?

Where do you normally access contraceptive services? Why this particular place?

Have you accessed contraceptive services from other places?

Do you know of any other places where you can access contraceptive services?

Tell me about the last time you accessed contraceptive services.

CHECK TAPE

The range of contraceptives available has dramatically increased in the past few years. What are your attitudes towards long term and short term contraceptives?

Prompts:

Some types of contraceptives are only 99% effective in preventing pregnancy – how do you feel about this?

Some types of contraceptives have certain side effects – how do you feel about this?

Do you think some people are unaware of certain types of contraception available, especially long-term methods?

Do you think there is a fear associated with implanted methods of contraception?

In your opinion, what is the most commonly used method of contraception?

Would you ever consider using implanted contraceptive methods? If no, why?

What do you think is the general reasoning behind using contraceptives?

CHECK TAPE

How do you feel about the different types of contraception that are currently available in the UK?

Prompts:

Do you feel you can make a better decision after knowing the number of services available?

Do you think all these contraceptives are easily accessible to you?

Were you aware of what was available the last time you accessed contraceptive services?

How do you feel about the different places where contraceptive services are offered?

CHECK TAPE

How do you feel about accessing contraceptive services through charity organisations such as homeless shelters?

Prompts:

How do you feel about trained staff being present at homeless shelters offering contraceptive services (long and short term)?

How do you feel about women being advised about their reproductive health at homeless shelters?

Have you got any further thoughts around contraceptive services?

Do you have any questions for me?

END TAPE

Conclusion:

- Collect demographic data on the interviewee.
- Ask the participant if they wish to receive a summary of the study findings.
- Give the participant the £5 food voucher.
- Thank the participant for their time.
